# Cardiac sarcoidosis

**DOI:** 10.1016/j.ero.2025.12.014

**Published:** 2026-01-21

**Authors:** Luca Arcari, Giovanni Camastra, Federica Ciolina, Luca Cacciotti

**Affiliations:** 1 Cardiology Department, Madre Giuseppina Vannini Hospital, Rome, Italy; 2 Radiology Department, Madre Giuseppina Vannini Hospital, Rome, Italy

A 59-year-old man presented to the emergency department because of dyspnea. The patient had mild pulmonary crackles and leg swelling. Electrocardiogram (Fig 1A) showed sinus rhythm with first-degree atrioventricular block and left bundle branch block. Transthoracic echocardiography revealed a hypertrophied and dilated left ventricle (LV) with severely reduced LV ejection fraction (LVEF, 25%). Nt-pro-BNP was 6045 pg/mL, and troponin T 71 pg/mL (normal value < 14 pg/mL) without a significant rise during in-hospital stay. Coronary angiography showed unobstructed coronary arteries. At cardiac magnetic resonance (CMR), late gadolinium enhancement (LGE) imaging revealed multiple scars with a nonischemic pattern (Fig 1, arrows in panel B). Specifically, LGE had variable transmural extension but often involved all the wall thickness in affected segments, and involvement of the basal interventricular septum was prominent (Fig 2, arrows in panel A). Concomitant myocardial edema with increased T2 mapping values as compared with in-center normal values [1] was noted, more evident in areas with LGE such as the mid-ventricular anterior wall (Fig 2, arrowheads in panels B and C, T2 mapping 68 ms shown in panel B). Native T1 mapping values were globally elevated due to the presence of both fibrosis and edema. A fluorine-18 fluorodeoxyglucose positron emission tomography (FDG-PET) showed increased contrast uptake of pulmonary lymph nodes (Fig 1, arrowheads in panel C) and the heart (Fig 1, arrowheads in panel D), matching LGE areas observed at CMR. What is the diagnosis?

**Figure 1:**
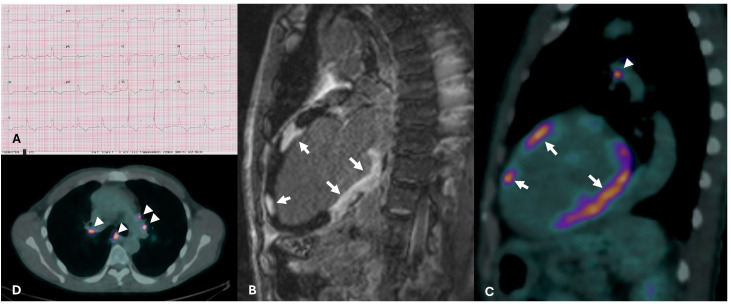
Electrocardiogram (panel A) showing sinus rhythm with first-degree atrioventricular block and left bundle branch block. Cardiac magnetic resonance (CMR) imaging showing late gadolinium enhancement (LGE) imaging with multiple scars with a nonischemic pattern (arrows in panel B). Fluorine-18 fluorodeoxyglucose positron emission tomography (FDG-PET) showing increased contrast uptake of pulmonary lymph nodes (arrowheads in panel C) and the heart (arrowheads in panel D), matching LGE areas observed at CMR.

**Figure 2:**
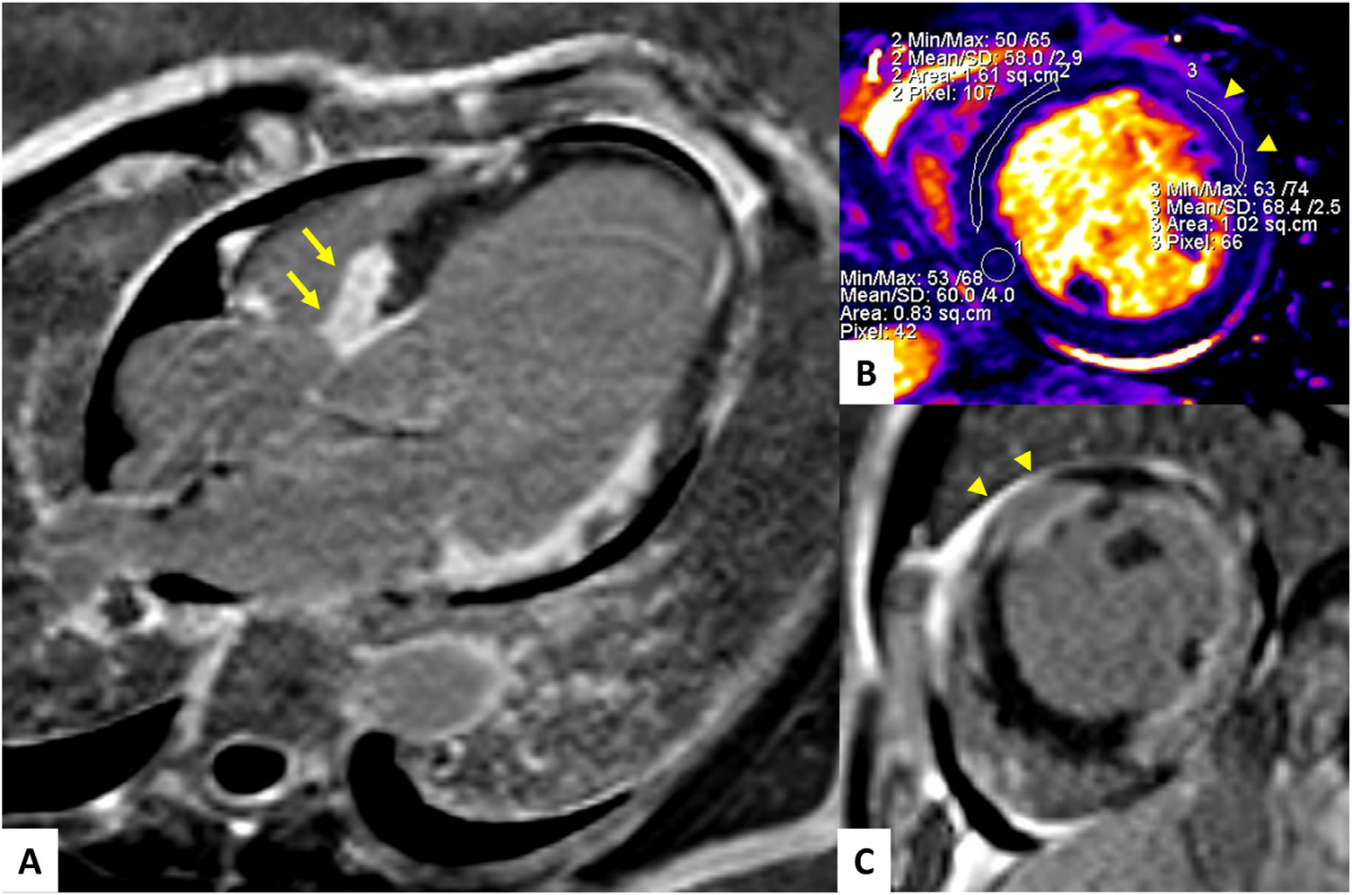
Cardiac magnetic resonance (CMR) imaging, late gadolinium enhancement (LGE) sequence, 4-chamber view showing multiple involved areas with variable transmural extension but often involving all the wall thickness in affected segments and prominent involvement of the basal interventricular (arrows in panel A). CMR imaging, T2 mapping sequence, mid-left ventricular short-axis view showing myocardial edema more evident in the anterior wall (68 ms as shown in panel B); on the same view, LGE imaging shows myocardial scarring corresponding to more edematous areas (arrowheads in panel B).

The clinical presentation and imaging findings were consistent with highly probable cardiac sarcoidosis, although a definite diagnosis cannot be established given the absence of histologic confirmation in our case [2,3]. Some serum biomarkers of cardiac sarcoidosis were not available, including ACE (Angiotensin-Converting Enzyme) and IL-6 (Interleukin) values. Serum calcium level was within normal range (9.3 mg/dL). The presence of multiple and heterogeneous myocardial scarring coupled with signs of active inflammation is typical of the disease [2]. This pattern is notably different from that observed in classic postviral myocarditis, who often have subepicardial involvement and rarely extensive transmural extension. Given nonspecific features of CMR findings and when biopsy should not be available, FDG-PET should be used to corroborate the diagnosis and assess for potential extracardiac involvement that must be confirmed by biopsy to obtain a definite diagnosis [3]. In this patient, the pathognomonic imaging findings including extracardiac uptake at FDG-PET suggested a “highly probable” cardiac sarcoidosis; however, in cases with more nuanced organ involvement [2], the integration of clinical, laboratory, and, ideally, histologic data are mandatory [3]. Immunosuppressive and heart failure therapies were started; however, because of a lack of LV function recovery, cardiac resynchronization therapy with defibrillator (CRT-D) was implanted. At follow-up after CRT-D implantation, improvement of symptoms was noted along with a reduction of NT-pro-BNP to 1256 pg/mL; however, cardiac volume and function only slightly improved and LVEF was 35%. Cardiac sarcoidosis is often underdiagnosed. CMR and subsequent nuclear imaging should be requested in the presence of unexplained LV hypertrophy/dysfunction associated with conduction disturbances.

## CRediT authorship contribution statement

**Luca Arcari:** Writing – review & editing, Writing – original draft, Visualization, Methodology, Investigation, Conceptualization. **Giovanni Camastra:** Writing – review & editing, Investigation, Conceptualization. **Federica Ciolina:** Writing – review & editing. **Luca Cacciotti:** Writing – review & editing, Supervision.
